# An Embedded Trace Redistribution Layer with Rounded-Bottom Cu Geometry and Ti Capping for Enhanced Electromigration Reliability

**DOI:** 10.3390/mi17050604

**Published:** 2026-05-14

**Authors:** Wonchul Do, Jeongmin Ju, Minjin Kim, Insoo Choi, Sanghyun Jin, Minkeon Lee, Hyeonho Yang, Jinho Jeong

**Affiliations:** 1 Global R&D Center, Amkor Technology Korea, Inc., Incheon 21991, Republic of Korea; wonchul.do@amkor.co.kr (W.D.); jeongmin.ju@amkor.co.kr (J.J.); minjin.kim@amkor.co.kr (M.K.); insoo.choi@amkor.co.kr (I.C.); sanghyun.jin@amkor.co.kr (S.J.); minkeon.lee@amkor.co.kr (M.L.); hyeonho.yang@amkor.co.kr (H.Y.); 2 Department of Electronic Engineering, Sogang University, Seoul 04107, Republic of Korea

**Keywords:** embedded trace, redistribution layer (RDL), electromigration, titanium barrier, reliability

## Abstract

This paper presents the electromigration (EM) performance of an embedded trace redistribution layer (ETR) in which the Cu trace features a rounded-bottom cross-sectional geometry and is encapsulated by a Ti barrier layer except for the top surface, with an optional top-side Ti cap. The ETR (with and without top-side Ti capping) and the conventional semi-additive-process (SAP) redistribution layer (RDL) are comparatively evaluated in terms of EM reliability. The ETR demonstrates a marked lifetime improvement compared with the SAP RDL. Notably, the Ti-capped ETR exhibits a minimal resistance increase in less than 10% even after a test duration of 4000 h. We discuss the key contributing factors and underlying mechanisms that support these improvements. Transmission electron microscopy (TEM) combined with atomic-percentage mapping confirms the effectiveness of Ti capping as a Cu diffusion barrier, showing continuous Ti coverage and no observable Cu diffusion. Electro-thermal simulations co-locate predicted thermal hot spots with experimentally observed open-failure sites, highlighting temperature-driven EM acceleration and the necessity of a barrier to suppress Cu–polymer interfacial oxidation. Stress simulations, together with EM failure analysis, indicate that the rounded-bottom Cu geometry alleviates local stress concentration and stress gradients, thereby creating conditions favorable for enhanced EM resistance.

## 1. Introduction

Chiplets and heterogeneous integration using organic-based redistribution layers (RDLs) are becoming mainstream packaging solutions for enhancing device- and system-level performance in artificial intelligence (AI) and high-performance computing (HPC) applications [1,2,3]. In both RDL interposers and silicon bridge-based interposers, RDL provides not only inter-chiplet interconnects and chiplet-to-high-bandwidth-memory (HBM) links but also power and ground distribution [4,5]. The semi-additive process (SAP) is widely used for RDL fabrication, whereas organic-based embedded trace RDLs (ETRs) utilizing a damascene process have been introduced to achieve enhanced scalability, reliability, and performance [6,7,8,9,10,11,12,13].

As RDL dimensions continue to scale down, reliability requirements become increasingly stringent. Because RDLs must handle higher data rates and current densities, Joule heating emerges as a limiting factor for the current-carrying capacity that must be maintained over the product lifetime, thereby raising electromigration (EM) reliability concerns. Therefore, mitigating EM-induced damage is essential [14,15,16].

In organic-based RDLs, one of the most influential factors affecting EM performance is the interaction between the Cu conductor and the polymer dielectric. During EM stressing, Joule heating inevitably occurs, and oxidation at the Cu–polymer interface promotes void formation and delamination, which reduces the effective cross-sectional area of the Cu. This reduction induces increased current crowding, further accelerating Joule heating in a positive feedback loop that ultimately leads to local Cu melting and dielectric breakdown at specific locations. These observations highlight the critical importance of preventing Cu oxidation to enhance EM reliability [17,18,19,20].

To improve the EM reliability of organic-based RDLs, several strategies have been investigated in the literature. One approach involves the use of inorganic capping layers (e.g., SiCN, Si_3_N_4_/SiO_2_, and TiO_2_) on SAP RDLs to suppress Cu oxidation and diffusion [21,22,23,24]. A second strategy focuses on Cu microstructure engineering, where nanotwinned or nanocrystalline Cu has demonstrated improved EM resistance [25,26,27]. A third approach involves developing more EM-compatible organic dielectrics or using organic capping materials—such as molding compounds—to cover the RDL, thereby blocking moisture penetration and Cu–polymer interfacial reactions [28,29]. Finally, adopting a damascene-type Cu architecture, in which the sidewalls and bottom surfaces are confined by diffusion barriers, has also been shown to improve EM reliability [30].

**Figure 1:**
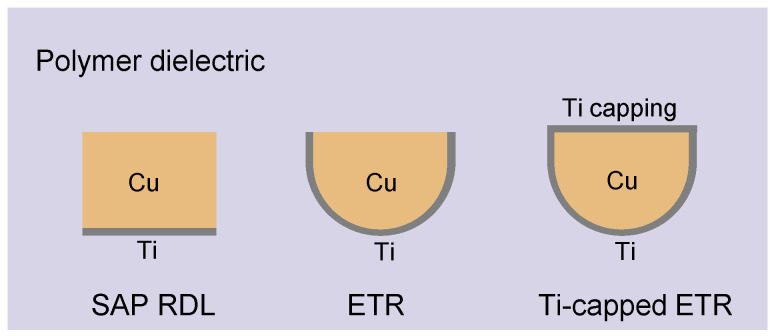
Cross-sectional schematic comparison of the three RDL structures evaluated in this study.

Approaches such as inorganic/organic capping and Cu microstructure engineering for improving EM reliability require careful consideration of the structural integrity of multilayer RDLs as well as package- or system-level reliability when materials with dissimilar properties are introduced [31]. In addition, most prior studies on EM reliability enhancement have focused on SAP RDLs and given little attention to the cross-sectional geometry of the Cu conductor. To the best of our knowledge, no systematic EM study of a rounded-bottom Cu cross-section in organic-based ETRs has been reported. This work investigates the EM performance of a high-density organic-based ETR with a round-ed-bottom geometry formed using a grayscale lithography method, in which locally modulated UV intensity is irradiated onto the photosensitive dielectric. After development, the resulting opening reflects the UV intensity profile, and subsequent dielectric curing induces shrinkage, leading to the rounded geometry [30,32]. This provides an integrated understanding of geometry-driven stress effects, full barrier encapsulation, and electro-thermal failure physics in organic-based RDLs. The rounded geometry reduces current crowding that typically occurs at sharp corners, thereby leading to lower signal transmission loss, as reported in our previous work [30]. This reduction in current crowding can mitigate localized Joule heating and thereby contribute to improved EM reliability. In addition, the rounded geometry provides a stress smoothing effect, which will be discussed later in this paper. We compare the EM performance of this ETR with a conventional SAP RDL and apply Ti capping to the top Cu surface to assess any further improvement. Ti capping was selected because it provides mechanical buffering between Cu and the organic dielectric, offers good electrical connectivity such that no separate opening process is required unlike inorganic capping layers, and is already widely used in semiconductor manufacturing, thereby not requiring substantial additional investment or specialized process development. Figure 1 schematically illustrates the three RDL structures evaluated in this study.

Section 2 describes the EM test vehicle fabrication flow, RDL cross-sections, current settings derived from Joule-heating calibrations, and evaluation and analysis procedures. Section 3 presents in situ resistance measurements, mean time to failure (MTTF), cumulative failure probability, and failure analysis results. Section 4 discusses the effectiveness of Ti capping based on TEM analysis, the temperature effects inferred from the co-location of electro-thermal (Joule-heating) simulation-predicted hot spots with observed failure sites, and the mitigation of local stress by the rounded-bottom geometry and Ti capping, supported by stress simulations and an auxiliary high-temperature storage (HTS) test.

## 2. Experimental

### 2.1. EM Test Package and RDL Fabrication

**Figure 2:**
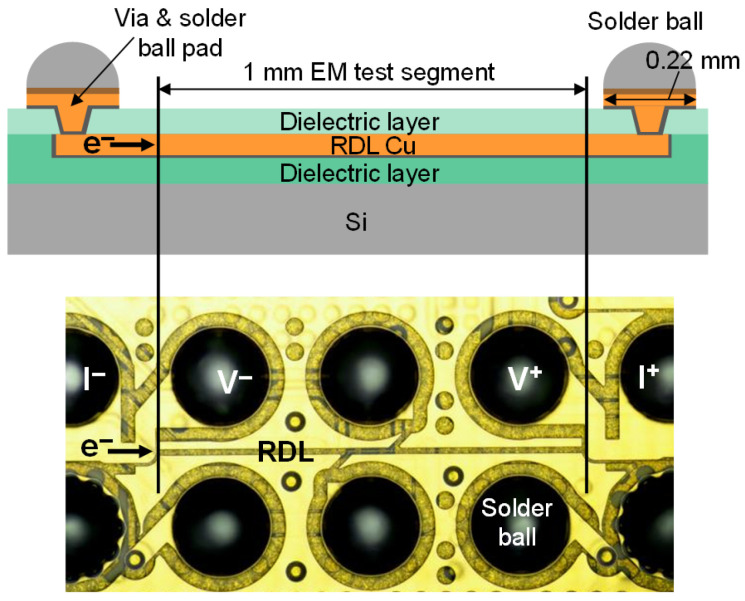
Stack-up schematic and plan-view image of the EM test package.

To fabricate the EM test packages, RDL fabrication and ball grid array (BGA) ball attach were carried out on 12-inch Si wafers. The wafers were subsequently singulated into individual package units by dicing and then surface mounted on daughter boards, which were connected to an EM test board through sockets. The test boards were placed in an oven as a part of the EM test system, which is equipped with current sources, a measurement unit, and a data monitoring system. All 12 samples for each RDL type were tested simultaneously under identical conditions. By adopting this wafer-level packaging approach, unnecessary connections and interfaces that may cause unexpected failures were minimized. The EM test package size is 8.5 mm × 8.5 mm. The Sn–Ag–Cu BGA diameter is 0.2 mm. The BGA pad connected to the RDL has a Ni–Au finish, and its diameter is 0.22 mm. As depicted in Figure 2, the EM test line, which is 1 mm long, is terminated at both ends with substantially wider Cu traces to ensure that failure occurs exclusively within the intended region.

**Figure 3:**
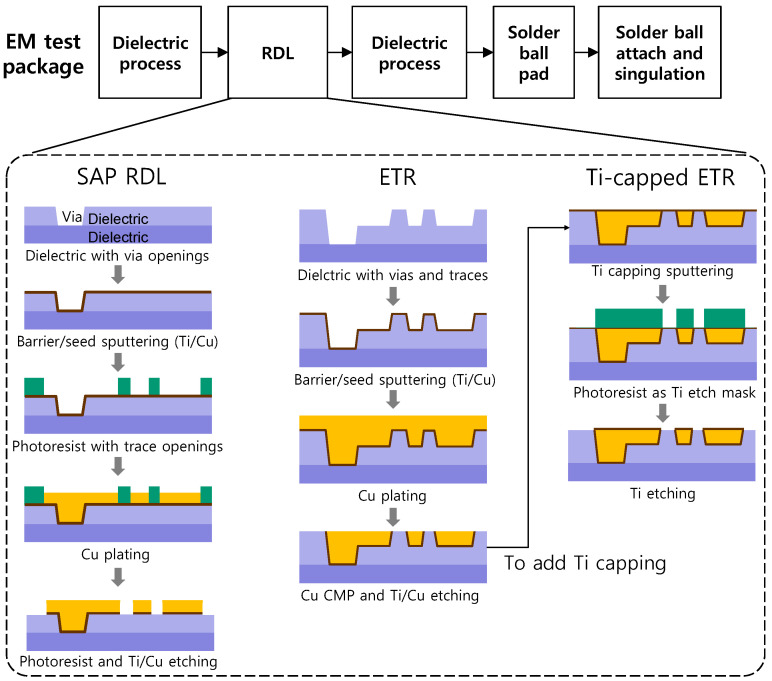
Fabrication flows of the EM test package and the corresponding processes for the three RDL structures.

Figure 3 presents the process flow for fabricating the three different RDL structures. Two 3 µm-thick dielectric passivation layers—one below and one above the RDL conductor—were formed to fully encapsulate the trace. A phenolic-based, positive-tone polymer with i-line (365 nm) sensitivity was used as the dielectric and cured for 2 h at 250 °C. The Ti barrier and Cu seed layers were deposited by sputtering with thicknesses of 50 nm and 100 nm, respectively. To fabricate the SAP RDL, Cu was electroplated inside the photoresist opening for 5.5 min at a current density of 30 mA/cm^2^. After Cu plating, the photoresist was removed, and Ti/Cu wet etching was carried out. For the ETR, vias and trenches for the traces were formed within the photosensitive dielectric, and Cu was electroplated into them for 10 min at 30 mA/cm^2^. Chemical mechanical polishing (CMP) was then performed to planarize the Cu overburden, followed by Cu and Ti wet etching steps. To add the Ti cap, the same 50 nm-thick Ti layer was sputtered, and unnecessary areas were etched away using a photoresist wet-etch mask. The target cross-sectional area of the RDL conductor was set to approximately 6 µm^2^, reflecting recent high-density routing requirements (e.g., SAP RDL with 2 µm width and ~3 µm Cu thickness) [2].

### 2.2. EM Stress Conditions, Calibration, and Measurement

The EM tests were performed under carefully controlled conditions to ensure equivalent current density (1.5 × 10^6^ A/cm^2^) and temperature (170 °C). The test conditions were determined based on three considerations. First, referring to previously reported EM studies [17,18,21], the conditions were selected to avoid excessively severe or overly mild stressing conditions, thereby ensuring that failures would occur within a practical timeframe ranging from several tens to several thousand hours. Second, the current density was chosen to reflect the increased current density requirements associated with RDL down scaling, while still providing accelerated testing conditions. Third, the test temperature was selected to prevent premature failure in other components, such as solder joints, due to thermally induced degradation. To compensate for differences in cross-sectional area, forcing current and temperature calibrations were performed, as summarized in Table 1. First, the actual cross-sectional areas of the fabricated RDLs were measured, and the applied currents were adjusted accordingly to achieve the same current density. Second, to set the EM test chamber temperature, Joule heating induced by the applied current density was determined using the temperature coefficient of resistance (TCR) obtained from resistance measurements over a range of temperatures under a low current. The temperature rise attributed to Joule heating is given by [33,34]:

(1) $$R(T)=R_{0}\left[1+\alpha (T-T_{0})\right]$$

where *R*(*T*) is the resistance at temperature *T*, *R*_0_ is the resistance at the initial temperature *T*_0_, and *α* is the temperature coefficient of resistance.

The percentage increase in resistance was measured for 12 RDL samples of each type during the EM test until an open circuit occurred. The initial resistances under EM stress conditions were 4.3–4.6 Ω for the SAP RDL, 3.1–3.3 Ω for the ETR, and 2.9–3.1 Ω for the Ti-capped ETR. The time-to-failure (TTF) was defined as the time at which the resistance increased by 20%, and the MTTF was determined based on a lognormal distribution.

## 3. Results

### 3.1. Fabricated RDL (Time-Zero Analysis)

**Figure 4:**
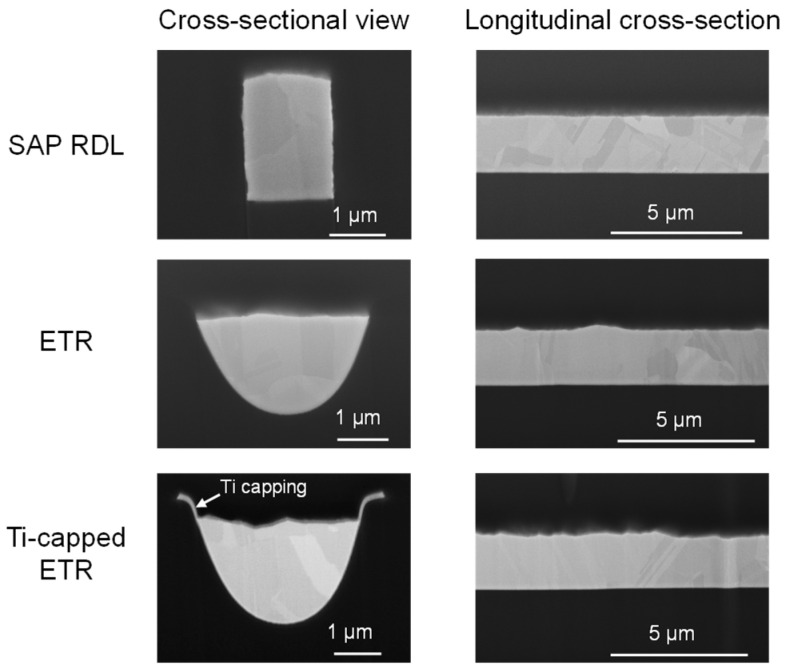
Cross-sectional FIB images of as-fabricated RDLs (time-zero condition).

Figure 4 shows the cross-sectional focused ion beam (FIB) images. The Ti-capped ETR exhibits a slightly recessed Cu top surface relative to the lateral Ti lines. This profile results from (i) Cu recession in the dielectric trench during Cu wet etching performed after the CMP step, rather than from limitations in CMP precision, and (ii) blanket Ti deposition followed by Ti wet etching using a photoresist mask intentionally wider than the Cu width to ensure full coverage and to account for overlay tolerance. A rougher surface is observed in both the ETR and the Ti capped ETR compared to the SAP RDL. The increased surface roughness is attributed to the longer Cu etching time applied after CMP in the ETR process. In the ETR process, post CMP Cu wet etching is performed to remove both residual plated Cu and the seed Cu layer used for electroplating, whereas in the SAP RDL process, Cu etching is performed only for seed layer removal.

**Figure 5:**
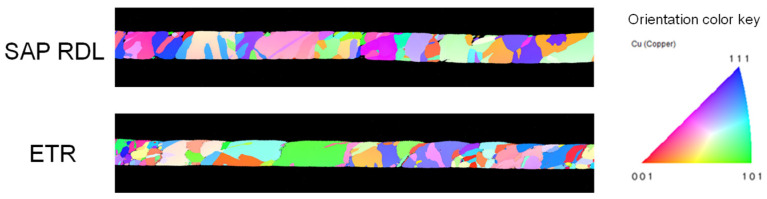
Longitudinal cross-sectional EBSD inverse pole figure (IPF) maps of the SAP RDL and ETR.

Electron backscatter diffraction (EBSD) was conducted to rule out microstructural artifacts, as the objective of this study is to assess the effect of the Cu cross-sectional geometry and the role of Ti as a diffusion barrier on EM performance. As shown in Figure 5, both the SAP RDL and the ETR exhibited polycrystalline microstructures with no dominant grain texture, and their average grain sizes were comparable (SAP: 2.35 µm; ETR: 2.13 µm).

### 3.2. Resistance Change and EM Lognormal Lifetime Distribution

**Figure 6:**
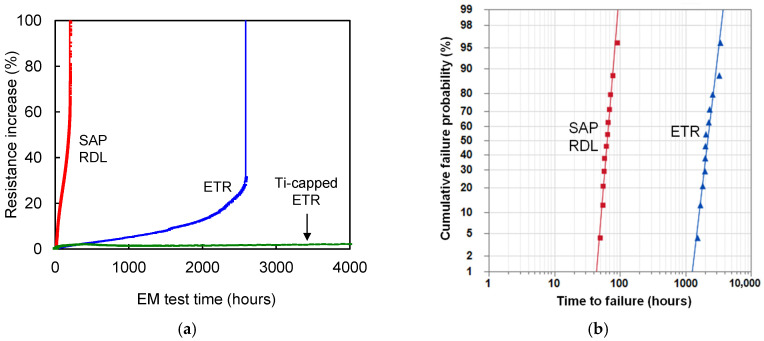
(**a**) Measured resistance change (%) of representative RDL test lines during EM stressing. (**b**) Cumulative failure-probability plots of the time to a 20% resistance increase.

Figure 6a shows the percentage increase in resistance for representative samples of the SAP RDL and the ETR. Across the 12 tested specimens, the time to a 20% resistance increase for the SAP RDL and the baseline ETR is comparable to their respective MTTFs, whereas the Ti-capped ETR exhibits only a median increase at 4000 h, because no sample exceeded 10% within the test window. The ETR exhibits a much slower resistance rise, and full Ti enclosure by adding a top-side cap markedly enhances EM performance. The MTTF, defined by a 20% resistance increase criterion, was 2202.85 h for the ETR—34.2× longer than the 64.46 h of the SAP RDL. Figure 6 was replotted from our previous work [30] for the SAP and baseline ETR curves, and the Ti-capped ETR curve was newly added in this study for direct comparison.

### 3.3. Failure Analysis

**Figure 7:**
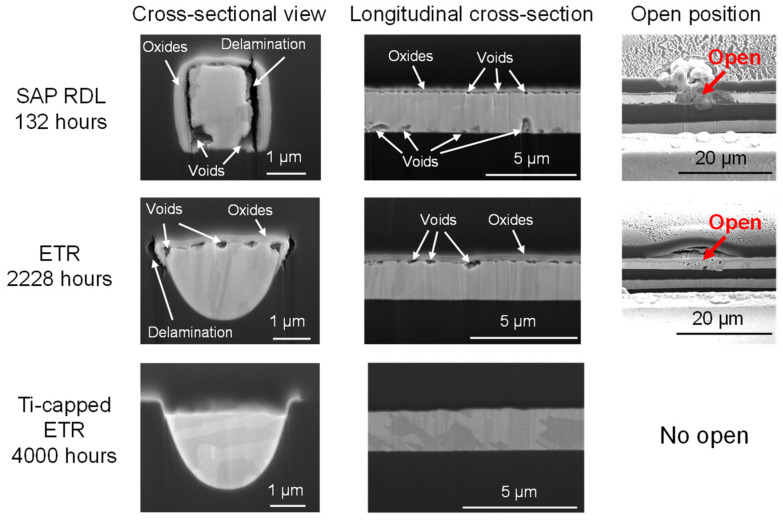
Cross-sectional FIB images of the SAP RDL, ETR, and Ti-capped ETR after EM testing.

Figure 7 presents the failure analysis results obtained using FIB. As expected, the cross-sections of the SAP RDL show large voids in the bottom corners and delamination at the Cu–dielectric interface, where Cu directly contacts the polymer dielectric. The longitudinal cross-section shows larger voids formed at the Cu-Ti interface. In contrast, in the ETR, Cu oxidation and voids are observed on the top surface, which lacks a Ti barrier, while the other surfaces remain intact. No evidence of Cu oxidation and voids is observed in the Ti-capped ETR. The locally molten traces are identified as the primary cause of the exponential increase in resistance of both RDLs.

## 4. Discussions

As shown in the results, the ETR exhibits a slower resistance increase, a longer EM lifetime, and suppressed voiding compared to the SAP RDL. To determine the underlying factors and mechanisms of these improvements, we performed follow-up analyses and simulations, including TEM/EDS of interfaces, electro-thermal hot-spot mapping, stress modeling, and HTS testing.

### 4.1. Effects of Ti Capping

By adding a top-side Ti cap, the embedded trace can be fully encapsulated with a Ti barrier. To verify the effect of Ti as a diffusion barrier, TEM analysis was performed on the time-zero and post-4000 h EM-tested specimens. The top-side Ti cap must be continuous and seamlessly connected to the ETR’s existing Ti barrier. Cross-sectional TEM–EDS mapping confirms a continuous Ti path from the sidewalls to the top surface, with no detectable breaks. The O signal follows the outer contour of Ti and becomes more pronounced after 4000 h of EM stressing, indicating increased formation of TiO_x_ at the cap/dielectric interface. This TiO_x_ layer provides good adhesion to the organic polymer by forming strong chemical bonds and blocks diffusion paths, thereby preventing Cu-ion penetration into the polymer [35,36,37,38].

**Figure 8:**
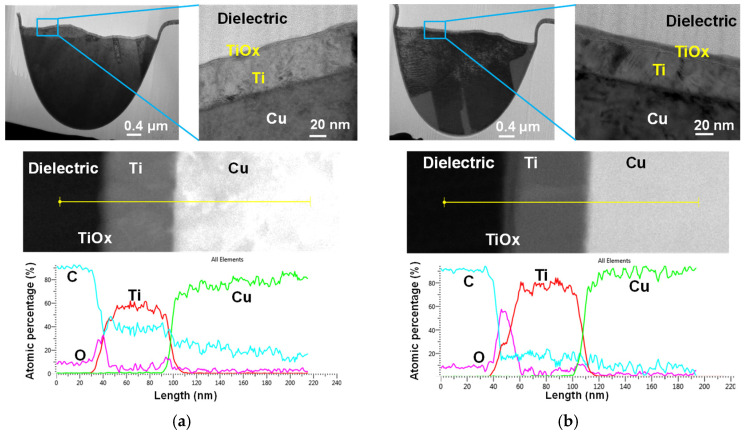
Cross-sectional TEM images and atomic-percentage line profiles of the Ti-capped ETR: (**a**) time-zero condition and (**b**) after 4000 h of EM stressing.

**Figure 9:**
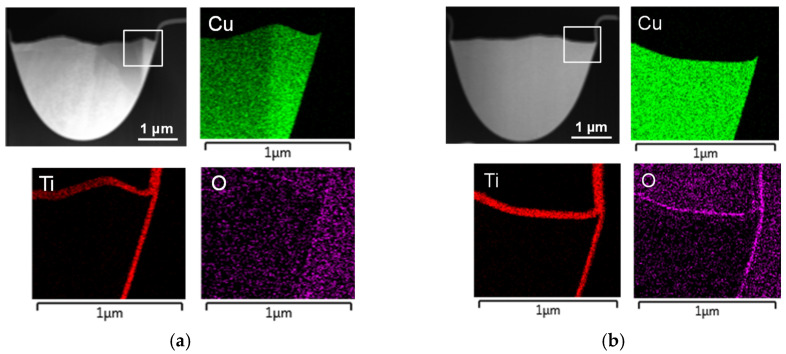
TEM elemental maps of the Ti-capped ETR: (**a**) as-fabricated (time-zero) sample and (**b**) sample after 4000 h of EM testing.

Figure 8 shows cross-sectional TEM images and EDS line scans for atomic-percentage profiles. A thicker TiO_x_ layer is observed in the sample after 4000 h of EM stressing. These observations are consistent with the elemental mapping results in Figure 9. The Ti signal rises from near zero on the dielectric side, reaches a plateau across the Ti layer, and then falls toward the Cu side. An O peak at the dielectric/Ti boundary confirms the formation of Ti oxide. Cu remains near zero across the Ti layer and increases only after the Ti profile drops, indicating no Cu diffusion through the Ti layer.

### 4.2. Co-Location of Simulated Joule-Heating Hot Spots and Observed Failure Sites

**Figure 10:**
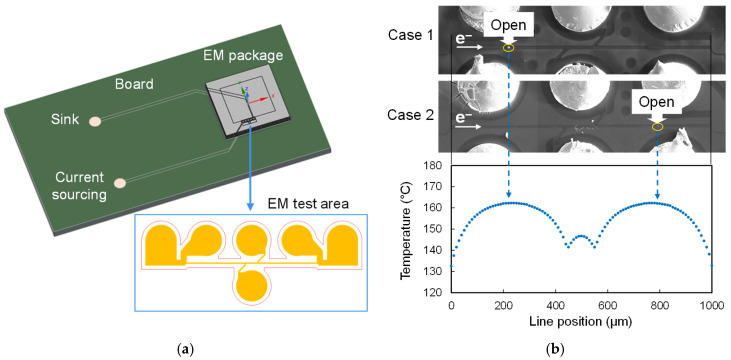
(**a**) Overall electro-thermal simulation model, (**b**) correlation between experimentally observed open-failure locations and simulated temperature rise along the EM test line.

Joule heating and the resulting temperature distribution were analyzed using an electro-thermal simulation tool (Ansys Mechanical 2026 R1). The simulation results indicate that the peak Joule-heating temperature reaches 162.3 °C, which is within ~4.5% of the actual EM test condition. The resulting temperature profile exhibits a double peak because two connections to the BGA pads near the midpoint of the EM test line provide localized Joule heating dissipation paths. Both peaks are located ~230 µm from either end of the 1 mm-long test line. Notably, these peak-temperature locations coincide with the open-failure sites observed in the EM tests (Figure 10b), confirming that the electromigration characteristics of polymer-dielectric RDL structures are strongly influenced by localized temperature rise. At Joule-heating hot spots, oxidation and void growth at the Cu–polymer interface reduce the effective Cu cross-sectional area, thereby accelerating current crowding. Therefore, suppressing Cu–polymer interfacial reactions using a barrier layer such as Ti in the ETR is critical for enhancing EM reliability. Furthermore, in widely adopted high-density RDL designs employing 2 µm-wide fine lines, the via cross-sectional area is typically more than an order of magnitude larger than that of the line. Consequently, EM-vulnerable regions are expected to occur at specific locations along the fine line and can be predicted through electro-thermal simulation, as validated by experimental observations. This correlation underscores the critical role of localized thermal gradients in determining EM reliability in polymer-dielectric RDLs.

### 4.3. Effects of Cross-Sectional Geometry and Ti Capping on Thermal-Stress

As the structural complexity of advanced packages increases owing to the co-existence of various materials and feature-size scaling, stress is becoming a more influential factor in EM failure. Larger stress gradients increase the atomic driving force, and the stress-driven atomic flux is given by

(2) $$J_{\sigma }=-\frac{DC\Omega }{kT}\nabla \sigma$$

where D is the diffusivity, C is the concentration of mobile species such as atoms and vacancies, Ω is the atomic volume, k is Boltzmann’s constant, T is the absolute temperature, and σ is the hydrostatic stress. Therefore, any geometry that promotes higher local flux divergence can increase the probability of void nucleation [22,39,40,41]. Since our objective is to study the effects of cross-sectional geometry on EM performance rather than plan-view layout design, we focused on the local stress distribution and analyzed thermally induced stress behavior locally at the Cu cross-section.

**Figure 11:**
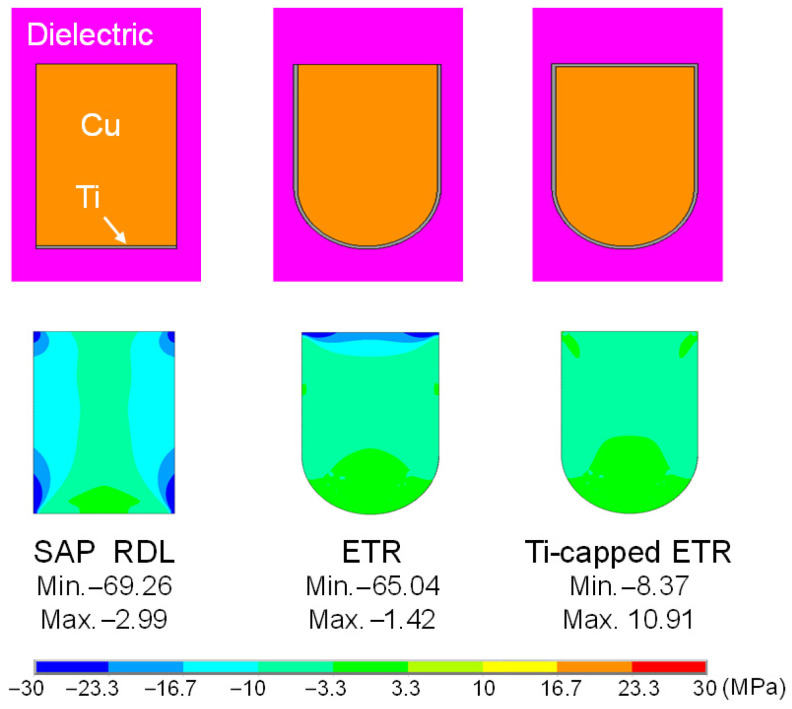
Cross-sectional models for stress analysis of Cu traces with three RDL cross-section types and simulated third-principal-stress contours within the Cu traces at 170 °C.

Figure 11 shows the finite element analysis (FEA) model (Ansys Mechanical 2026 R1) for the RDL stress simulation to examine how the conductor cross-sectional shape affects the local stress distribution inside the conductor. In our simulation, the polymer cure temperature (250 °C) is taken as the stress-free reference temperature. Since the EM test temperature (170 °C) is lower, the polymer—with a higher coefficient of thermal expansion (CTE)—tends to shrink relative to Cu, imposing compressive stress on the Cu trace. To visualize compression-dominated hot spots and their cross-sectional gradients, we plot the third principal stress. High compressive stress concentration—and thus stress gradients—are observed at all four corners of the SAP RDL, whereas only the top two corners show concentration for the ETR. This indicates that the rounded shape helps reduce stress concentration and gradients. A high stress gradient drives atoms from the compressive corner region toward nearby tensile zones, leaving vacancies at the corner and thereby promoting void nucleation [40]. Adding Ti capping further reduces the stress gradient even at the top corners. The stress gradients at 170 °C are 66.27, 63.62, and 19.28 for the SAP RDL, the ETR, and the Ti-capped ETR, respectively. It appears that Ti capping makes the top boundary more uniform and stiffer, which mitigates polymer-induced asymmetric constraint under the temperature difference between 250 °C and 170 °C, thereby reducing corner stress and gradients.

**Figure 12:**
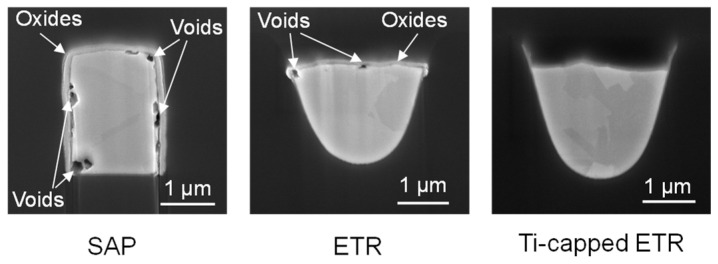
Cross-sectional FIB images of the RDL structures after HTS at 150 °C for 500 h.

To investigate whether thermally induced stress gradients associated with conductor cross-sectional geometry contribute to an increased probability of void nucleation and growth, a HTS test at 150 °C without applying any current was performed in accordance with the JEDEC standard (JESD22-A103 [42]). As shown in Figure 12, Cu oxide is observed in regions where no Ti barrier layer is present—in the SAP RDL and in the ETR without a Ti cap—whereas no Cu oxidation is detected in the Ti-capped ETR. In the SAP RDL, voids are observed at the bottom-left and top-right corners, while additional voids appear at the top-left corner and near the top-center region. The HTS and stress-simulation results suggest that a rounded geometry and fully Ti-encapsulated Cu together constitute favorable conditions for improving EM resistance.

Based on the Ti capping effects we analyzed in Section 4.1 and this section, there is potential for improved EM performance in SAP RDLs if the same Ti capping scheme is applied. However, EM performance is influenced not only by the barrier role of Ti but also by stress distribution arising from the geometry and defects introduced during the fabrication process. In the case of SAP RDLs, stress concentration associated with sharp corners may still occur, and incomplete Ti coverage at the bottom corner regions is possible due to seed layer undercut during processing [9,21]. Considering these factors, the ETR structure still possesses advantages that can lead to superior EM performance.

## 5. Conclusions

This study shows that an organic-based ETR with an as-formed rounded-bottom Cu cross-section achieves a 34.2× increase in EM lifetime over a conventional SAP RDL. With Ti capping, the EM reliability margin is further enhanced, with ≤10% resistance increase within the 4000 h window and no failures observed. These gains arise from the dual actions of Ti-enabled Cu diffusion blocking and cross-section-driven mitigation of local stress concentration and stress gradients, as supported by TEM and FIB analyses, electro-thermal and stress simulations, and failure-site observations. The results indicate that the proposed ETR architecture translates directly into an increased EM-limited current-carrying capacity for RDL interposers and silicon-bridge-embedded interposers in artificial intelligence and data-center applications. Future work includes the formulation of design-level EM guidelines by extracting the activation energy and current exponent.

## Figures and Tables

**Table 1:** Current and temperature calibration for the EM test.

RDL	Cu Cross-Section Area (μm^2^)	Applied Current (mA)	Joule Heating by Applied Current (°C)	EM Test Chamber Temperature (°C)
SAP	4.6	69.0	41	129
ETR	6.1	91.5	44	126
Ti-capped ETR	6.7	100.5	48	122

## Data Availability

The data presented in this study are available from the corresponding author upon reasonable request. Public sharing is restricted by company confidentiality obligations.
